# Motivational Influences Affecting Middle-Aged and Elderly Users’ Participation Intention in Health-Related Social Media

**DOI:** 10.3390/ijerph191811240

**Published:** 2022-09-07

**Authors:** Cong Cao, Dan Li, Qianwen Xu, Xiuyan Shao

**Affiliations:** 1 School of Management, Zhejiang University of Technology, Hangzhou 310023, China; 2 School of Economics and Management, Southeast University, Nanjing 211189, China

**Keywords:** motivation, middle-aged people, elderly people, health-related social media, participation intention

## Abstract

Social media provide users with multi-directional dialogue for creating and sharing health information that can effectively promote the self-management of health. In regard to the ‘greying’ trend in social media, most researchers have studied the health-related social media (HRSM) acceptance status and use behavior of middle-aged and elderly people, and have explored the role of HRSM in this group. However, the continuous participation of users is the key to the successful operation of HRSM, and is an essential prerequisite for the subsequent HRSM behavior habits of middle-aged and elderly people. Therefore, we aimed to explore what motivations drive the first use of HRSM among middle-aged and older adults, and the impact of their perception of HRSM, after personal use, on their intention to use it continually. In the study, we used the partial least squares structural equation model (PLS-SEM) to analyze data collected from online questionnaires. The results showed that a self-protection motivation and a social motivation promoted the initial participation of middle-aged and elderly individuals. In addition, these people experienced deeper levels of perceived usefulness and perceived entertainment after their initial participation. The results also revealed that these two perceptions could positively influence middle-aged and elderly individuals’ intention to continue with their participation. Our findings should help service platforms to better understand the needs of middle-aged and elderly users. This would help researchers and practitioners to gain a more complete understanding of the motivation of middle-aged and elderly people for participating in HRSM, and the related impacts this may have.

## 1. Introduction

According to research, population growth in most countries of the world has slowed down, and their fertility rates are low, with the result that, globally, there is an obvious trend towards social aging. Due to factors such as pension demands and environmental policies, the aging population has diverse effects on the economy, and the standard of living of the elderly has changed. Middle-aged and elderly people have become a significant part of economic consumption. Their savings rate has risen, and the expenditure on health care has ushered in faster growth [1]. For aged people, age-related disease burden rates decrease over time. Using relevant medical information and resources can help reduce the projected burden of population aging [2,3]. With the widespread popularization of the internet, everyone can now participate in social media, and social media aging has also become a global reality [4]. Studies show that the internet is the main source of information collection, and the number of middle-aged and elderly people who use social media to find and evaluate health information continues to increase [5]. Through virtual networks and communities, social media share and exchange electronic content, containing various forms of expression, expand social networks, and establish connections with other social groups [6]. Health-related social media (HRSM) are online media used to obtain health information regarding diet [7], exercise [8] and other aspects of health, such as online health communities and health-related social networking sites. Moreover, with the COVID-19 pandemic, digital solutions such as social media have become an important way of keeping in touch and obtaining information [9]; therefore, an exploration of social media has important practical significance.

A search of the current literature reveals that social media can play a positive role in health communication, disease prevention, and healthy lifestyle promotion, among other areas [7,8,10,11]. In terms of health education, Goodyear et al. [12] point out that while HRSM play a positive role among young people, the engagement of young people will be affected by information content, usage mode, and interactive function. These authors propose that the importance of social media as a health-related learning tool in the informal learning environment should be accepted and supported [13]. Several recent studies have called for social media to broaden their health education channels, improve access to health information, and improve the self-efficacy of users [14,15,16,17,18]. However, the use of HRSM can also introduce potential risks, as the information they contain may have considerable influence on individuals’ health concepts and behaviors [19]. Information presented on the platforms is often false or inaccurate [20], and the spread of false information has the potential to cause anxiety or panic, resulting in unhelpful behaviors and negative health consequences [21,22]. For example, Turner and Lefevre [23] found that the frequency of Instagram use was positively correlated with the occurrence of orthorexia and the severity of the symptoms. Middle-aged and elderly people frequently have smaller social capital in HRSM than younger people, and they are more likely to experience problems, such as the digital divide, low electronic health literacy, and loneliness, while some of this group are unable or unwilling to make full use of social media functions [24,25]. The use of HRSM may in fact undermine the confidence of middle-aged and elderly people, and aggravate negative emotions [4]. In contrast, Bernhardt et al. [26] analyzed the social characteristics of Facebook, LinkedIn, and Twitter, and revealed that the prudent use of social media can promote personal growth, and that its advantages and benefits far outweigh its risks. Social media is thus shown to have the potential to affect an aging population in different ways. Bearing in mind both the advantages and disadvantages, we conducted a study on whether middle-aged and elderly people can perceive benefits in using HRSM.

Although there is a clear trend towards social media users being older, the frequency with which social media are used declines with increasing age, and user growth is not evenly distributed among the various age groups [27,28,29]. Although the internet has become the main source of health information for middle-aged and elderly people, the number of older people using HRSM is still relatively small [5,28]. Some middle-aged and elderly people distrust social media, and do not believe the health information provided on the internet [30]; however, others blindly believe that all information on the platforms or in their social environment has the same, or even better, value and credibility as the diagnosis and treatment advice provided by professional medical institutions [31]. Due to a general lack of health literacy, the elderly are often not good at distinguishing true from false, when dealing with health information [32], and this makes the impact of HRSM on them more uncertain. Therefore, the potential positive effects of HRSM have not been maximized among the middle-aged and elderly. In our study we focused on how to position the role of HRSM correctly, so that it can make a positive contribution, and how to avoid or reduce any negative effects on the health of the middle-aged and elderly population.

To sum up, this study aimed to explore what motivates middle-aged and elderly people to start using HRSM; to identify changes in their perceptions after they had used it personally; and to study the influence of social media on their intention to continue using HRSM. The study differed from previous studies in that both initial participation and intention to continue to participate were studied, which filled a research gap. The research results provided valuable insights into the design of HRSM platforms. For example, platforms need to design mechanisms that are more suitable for middle-aged and older people; they need to expose middle-aged and older people to scientifically accurate health information; and they also need to design effective and interesting modes of communication and interaction.

This paper summarizes our search for relevant research into motivation to use social media. Based on the characteristics of middle-aged and elderly people, and on a related literature search, the paper proposed a research framework for a study that considered the two stages of HRSM use—initial participation and continued participation, for which we constructed corresponding research hypotheses, and designed an online questionnaire, based on scales in the existing literature. The questionnaire was used to collect relevant data, and partial least squares structural equation modelling (PLS-SEM) was used to evaluate and test the research model and hypotheses. Finally, the paper presents the corresponding research conclusions, with their theoretical and practical significance. In addition, the limitations of the study and future research directions are explained.

## 2. Research Framework

### 2.1. Literature Review

#### 2.1.1. Motivation Research into Social Media Use

Motivation is the factor that drives individual behavior when self-regulated, and it represents a key determinant of behavior [33]. In general, people are directed by both intrinsic and extrinsic motivation, and this has been verified by studies in various fields, such as the acceptance of new technology and green innovation [34,35,36,37]. In a study of social media use, motivation was generated by users’ health needs, and the functions provided. People use the internet to search for a variety of information [38,39]. The emergence of Web 2.0 has made health information widely available in society, through communication and interaction, and social media have become a potent source of health information [27]. Van de Belt et al. [40] point out that social media can be used to collect health information quickly and cheaply, and thereby to improve medical care. Gonzalez et al. [41] found that a positive experience (in terms of information quality and technology) with online health information searches can promote people’s use of HRSM. By contrast, improvements in their health status, and an increased perception of risk, can inhibit people’s interest in searching for information. In addition to providing necessary health information, HRSM have the general characteristics of other social media, such as interpersonal communication and interaction. Hardiker and Grant [42] conducted a summary analysis of the use factors of e-health services through inductive reasoning, including age, financial status, participation, and internet availability. On the technical level, they analyzed that, through the support of technical platforms (such as online communities and remote health care platforms), users also get emotional support, through interaction, and their contacts are deepened. Through a study of tweets on Facebook, Rahim et al. [43] showed that online reviews of hospitals can improve patient satisfaction, promote communication between doctors and patients, and thus help to build trusting relationships. Antheunis et al. [44] differentiated between user identities, and found that the main motivations of patients using HRSM were to gain knowledge, obtain social support, and exchange advice, while the main motivations of health professionals were marketing and communication with colleagues. Many studies have shown that the practicality and interactivity of HRSM help patients, hospitals, and healthcare professionals to participate in HRSM [45,46,47]. Therefore, based on the above studies, and on the health needs of the non-medical people using HRSM, this study divided the motivation to use HRSM into ‘protection motivation’ and ‘social motivation’, before exploring these further.

#### 2.1.2. Middle-Aged and Elderly People, Health-Related Social Media

Although young people are the main participants in social media [48], middle-aged and elderly social media users continue to increase [28]. Scholars have discussed the effects of social media on health, and empirical studies have shown that extensive participation in HRSM can positively promote the health of middle-aged and elderly users [49,50]. He et al. [51] conducted research from the perspective of social participation, and emphasized that social media can promote the formal and informal social participation of the elderly. Bonsaksen et al. [52] revealed that social media users of all age groups are associated with loneliness in the context of COVID-19. Research has shown that in the middle-aged and elderly groups, the more or the more frequently they use social media, the lower their sense of loneliness. Medlock, Eslami, Askari, Arts, Sent, de Rooij, and Abu-Hanna [28] found that the elderly will search for health information according to their own needs and preferences. Tennant et al. [53] found that older people who had used HRSM to obtain health information had higher electronic health literacy than those who had not, and that this played a role in their health education. Brunello et al. [54] pointed out that health education has an impact on the health behaviors of middle-aged and elderly people in the short term, and that it has an even deeper impact in the long term. When considering influencing factors, Khoo and Yang [55] found that both perceived social support and perceived constraints affected the executive functions of middle-aged and elderly individuals in relation to using social media. Parida et al. [56] revealed that technology user experience and technology attitude greatly affect elderly people’s use of social media for health-related activities.

Goodyear, Armour, and Wood [12] adopted a participatory and iterative research design. Through continuous and repeated qualitative analysis, they pointed out that the use of HRSM by young people is closely related to health information, and the positive and negative effects of such information have no positive tendency. Through the qualitative analysis of semi-structured interviews, Casanova et al. [57] found that SNS potentially alleviates the loneliness of the elderly, and is an effective support tool for social life. The study also emphasized that social contact with relatives and friends motivates the elderly to use SNS. Han et al. [58] analyzed personal and social contexts through semi-structured, in-depth interviews. Moreover, they found that communication quality, contact accessibility, perceived risk, and perceived health effects affected the behaviors of the elderly in their use of social media. The research also showed that social media could enhance cognitive engagement and improve health communication. There are various research methods for HRSM, including qualitative and quantitative research. At present, there is a relative wealth of HRSM research, identifying many influencing factors and mechanisms, but there are few studies on behavior evolution. The subject of this study was middle-aged and elderly people who had used HRSM. They were highly subjective and active. The composition of the study object was single. Furthermore, it was not possible to collect precise, relevant data during the data collection process. Interviews, field investigation, and other methods could not help to study the overall effect and directly draw conclusions. Therefore, a questionnaire survey method, that could obtain first-hand data, was selected. In addition, the questionnaire could satisfy both qualitative and quantitative research. In the early stage of the study, opinions, objectives, and other contents were collected, and inductive reasoning was carried out, in combination with existing research contents, to qualitatively describe middle-aged and elderly people’s motivation for using HRSM. Then, a PLS-SEM analysis of the structural model was carried out quantitatively, to verify the research hypothesis, and to improve the standardization and accuracy of the research.

On the whole, HRSM can play a positive role in the health management of middle-aged and elderly people, when used correctly. The participation of middle-aged and elderly people in HRSM is relatively low. Participation behavior is affected by health conditions, technology, and social demand, among other factors. Many scholars have explored the impact of social media use, but there is a lack of specific research into the motivation underlying participation behaviors in middle and late adulthood. Thus, after exploring the influence of initial motivation on initial participation, this study investigated the effect and influence of perceptions following participation. It then took the two kinds of perception after use as a new motivation to study intention with regard to continuous use by middle-aged and elderly users.

### 2.2. Hypotheses Development

**Figure 1:**
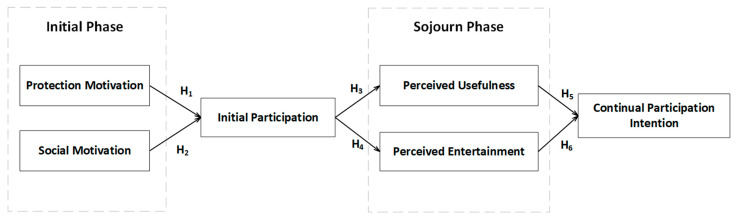
Research model.

After ascertaining the e-health behavior patterns of middle-aged and elderly people, we collected and collated relevant data and literature with the keywords ‘health-related’, ‘social media’, and ‘middle-aged and elderly people’. Based on the thinking mode of deductive reasoning, this paper explored the motivation deduction of middle-aged and older people using HRSM. After abstracting the questions from the background, qualitative questionnaires were used to collect the influencing factors and objectives. After summarizing and combining the existing theories and relevant studies, we classified the influencing factors and behaviors of HRSM. The participation of middle-aged and older people was divided into two stages, as shown in Figure 1. In the initial stage, middle-aged and older people were exposed to HRSM from the perspectives of understanding, curiosity, health, and social interaction. Based on the motivation theory, the initial participation was divided into two parts: behaviors based on protection motivation (such as health information searches and health product purchases) and behaviors based on social motivation (such as health knowledge sharing and watching live broadcasts). After the initial use, some groups intended continuous use. The intention to participate continuously was defined as the individual’s willingness to continue to use HRSM after the initial and multiple interactions. This was helpful for the follow-up study of extreme behavior or habitual behavior. Improvement of the continuous evolution of behavior intention should be related to the user’s use process (i.e., the staying phase); in order to correspond to the user’s functions (rationality and emotion), we defined the motivation as perceived usefulness and entertainment.

#### 2.2.1. Protection Motivation and Social Motivation

Initial participation refers to the very first time that an individual ever participates [59]. In this study, it refers to middle-aged and elderly people’s first-time use of HRSM functions, and their participation in activities such as browsing content and interacting. Behavior is dominated by motivation, and demand is a significant factor in generating motivation [60]. HRSM is mainly used to promote personal health, through communication and access to health-related information [48,53]. On this basis, we divided ‘motivation’ into ‘protection motivation’ and ‘social motivation’. ‘Protection motivation’ refers to the psychological tendency to protect the individual’s interests from damage [61]. In the present study, ‘protection motivation’ referred to an internal driving force to collect health information, seek professional advice, and learn health skills in order to protect health. In the locked-down environment during the COVID-19 epidemic, death anxiety, distress, and loneliness were all amplified [62]. Older adults have a higher mortality rate from COVID-19 than other age groups [63], and this has a profound impact on the death anxiety of the elderly, which directly affects their sense of loneliness [64]. These perceived threats are likely to stimulate and increase personal–protection motivation, and a strong protection motivation is likely to attract users to participate in social media communication [65].

‘Social motivation’ refers to gaining a sense of connection and belonging through interaction [66,67]. Studies point out that digital social networks can provide and disseminate social support (such as information or emotional encouragement) for users, without the limitations of time or geographical location [68]. In addition, many elderly people like using social media because social media provide a simple way to establish contact and keep in touch with family and friends [58]. In this study, social motivation was defined as the psychological tendency of middle-aged and elderly people to engage in a series of behaviors involving social interaction, such as meeting and making friends with interesting people. Previous studies have noted a correlation between social motivation and the use of social media by middle-aged and elderly users. Specifically, Rivas et al. [69] found that older users preferred message boards, for receiving general support and having discussions, more so than younger users under the age of 45 years. Kruse, Mileski, and Moreno [5] pointed out that social media use by the middle-aged and elderly is not only for communication between peers, but also for interaction with the younger generation, to increase connection and to reduce loneliness. As the health risks of middle-aged and elderly people increase with age, the self-protection health management offered by the health information channels of HRSM offers an effective way for them to acquire the medical care they need [70,71].

Based on this information, this study believes that middle-aged and elderly users will be influenced by both protection motivation and social motivation, to use social media channels, such as HRSM, and to participate in social interaction, namely:

**H1.**  *Protection motivation positively influences initial participation in health-related social media by middle-aged and elderly people.*

**H2.**  *Social motivation positively motivates middle-aged and elderly people to participate initially in healthy social media behaviors.*

#### 2.2.2. Perceived Usefulness and Perceived Entertainment

Perceived usefulness refers to users’ subjective beliefs about the benefits derived during their use of a system [72,73]. This study specifically shows the benefits of useful information and improved health status in middle-aged and elderly individuals, after using HRSM. Perceived entertainment refers to positive emotions—such as enjoyment, relaxation, and recreation—which users feel when using HRSM [74]. This study specifically referred to the entertainment emotions generated when middle-aged and elderly users use HRSM for the first time. It found that when users use social media, they form certain perceptions of its usefulness and entertainment value. Specifically, participating in HRSM can bring perceived benefits. With recent developments in technology, the health promotion model has changed, from providing threatening health-risk warnings, to offering encouraging self-management health guidelines. This new mode gently improves users’ sense of efficacy, and prompts them to meet their own needs [75]. In addition, social media can facilitate ‘patient empowerment’ through individuals learning and using various means (e.g., HRSM), to improve their health literacy, and to enjoy the health services offered. Furthermore, social media helps equip the users with self-regulatory skills. In this way, social media’s ability to meet the needs of users is effectively improved, as it simultaneously builds users’ understanding of the process [75,76]. Studies have proved that pages and interactions with an interesting design can enhance a user’s perceptions of entertainment when interacting with social media [77,78], and that this is conducive to improving the user’s satisfaction. This paper therefore argues that middle-aged and elderly people can enhance their perceptions of HRSM as being both useful and entertaining, during their initial participation, namely:

**H3.**  *The initial participation of the middle-aged and elderly in HRSM can enhance their perceptions of its usefulness.*

**H4.**  *After their initial participation in HRSM, middle-aged and elderly people have enhanced perceptions of it as entertainment.*

#### 2.2.3. Continued Intention to Participate

Intention to continue to participate refers to the degree of willingness to continue using functions and participating in activities [59]. A strong intention by the middle-aged and elderly to continue participating in HRSM will indicate that they are willing to use HRSM and spend more time on HRSM. The experience that users have, the first time they interact with social media, will affect their intention to use the social media again. Their subsequent participation is influenced by their perceptions of its usefulness, and their continued participation will be increased by greater perceived usefulness [79]. To be more specific, the functionality of social media directly affects its perceived usefulness. In turn, social media enhances user engagement through interaction with other users. In other words, the perceived usefulness of social media will positively affect users’ trust, while their sustained participation is promoted through the media’s page design and the presentation of information [73]. This understanding is verified in the field of technology acceptance: when using new technologies, the perceived usefulness of a new technology is both directly and indirectly related to the user’s acceptance of the technology, and the user’s willingness to continue using it [77]. If a design has good usability, its perceived usefulness will be increased, and a good user attitude towards it will be promoted [73].

In addition, previous studies have explored the influence of entertainment on the motivation of individuals to use information systems. They have found that the more entertaining the information system is, the more willing participants will be to use it [80]: perceived entertainment value positively affects users’ attitudes. The pleasure, or perceived entertainment, when interacting with a technology device, has been shown to be a key factor affecting the user’s intention to accept and continue to use the technology device [77,78]. Lee et al. [81] point out, in their research into websites, that perceived entertainment has a significant impact on website users, and that individual perceptions and enjoyment of website entertainment will improve users’ satisfaction with the website and their sustained participation. Hsu et al. [82] mention that the fun dimension of a website will affect the user’s perception of its value, which will then affect the user’s satisfaction and willingness to continue using it. In terms of social platforms, Curras-Perez, et al. [83] believe that users’ perceptions of entertainment impact their attitudes positively, affecting their willingness to recommend a social platform to others, and to continue using it. Based on this, we believed that the usefulness and entertainment value perceived by middle-aged and elderly individuals when using HRSM would positively affect their willingness to continue using the social platform, and we made the following assumptions:

**H5.**  *Perceived usefulness positively affects the willingness of middle-aged and elderly people to continue to participate.*

**H6.**  *Perceived entertainment value positively affects the willingness of middle-aged and elderly people to continue to participate.*

## 3. Materials and Methods

To explore the preconditions for middle-aged and elderly people to participate in HRSM, and their sustainable intentions to do so, this paper proposed a research model based on a literature research and on small-scale interviews. It then used PLS-SEM to evaluate the measurement model and structural model. The study tested the hypotheses mentioned above, by using a questionnaire-based survey. During the survey, the following principles were strictly observed: (1) participation was voluntary; (2) relevant information was recorded anonymously; (3) identifiable personal information was not collected, and strict data protection and storage policies were adopted; (4) participants were able to terminate or withdraw at any time; (5) the purpose of the study was disclosed in advance, and was open and transparent to the subjects; (6) there was no conflict of interests between the subjects and the researchers; (7) no data were recorded prior to the questionnaire being submitted; (8) information relating to the study was stored on one computer only, which could be accessed only by persons related to the study, and authorized by the supervisor, and the hard disk of the computer was encrypted.

### 3.1. Measurement Items

Based on the literature review and theoretical construction, we designed 7-point Likert scales to capture the measurements, based on established scales in the relevant literature. We invited 20 qualified volunteers in China to describe the relevant factors that may have influenced their HRSM participation. Through classification and combination, we determined our questionnaire. After completing the initial questionnaire design, we conducted a small-scale pre-test in related fields. A total of 30 questionnaires were collected, to check whether the semantic and grammatical expression of the options in the questionnaire could be understood easily, and whether its reliability and validity met requirements. At that point, we modified some expressions in the questionnaire, in accordance with the feedback received. Finally, a formal questionnaire was established.

We fully informed all potential respondents, regarding the study details, including the reason for the study, the purpose of the data, and the risks. All subjects were able to terminate or withdraw from the study at any time. After the submission deadline for the questionnaire, we counted all the data. We guaranteed that relevant information would be recorded anonymously during the whole process of the investigation, and that we would not collect information that could identify individuals. The data management for this study followed a strict management policy. All information was collected and stored on one computer, and the data were strictly encrypted. Only the research leader and technicians had access to the data. Information technology tools were used to translate the data, to organize the information to form a complete logical structure, and then to apply it reasonably.

The questionnaire consisted of three parts. Part A clarified the background, purpose, and use of the study. Part B collected demographic information about the respondents, such as gender, age, and education level. Part C presented questions on scales according to the existing literature, and measured six theoretical constructs: Protective Motivation; Social Motivation; Initial Participation; Perceived Usefulness; Perceived Entertainment; and Continual Participation Intention (as shown in Table 1). The respondents rated the statements on the questionnaire on a scale from 1 (=strongly disagree) to 7 (=strongly agree).

### 3.2. Sample

From 1 November 2021 to 31 January 2022, we distributed and collected the questionnaires in two ways: (1) offline: questionnaires were distributed in nursing homes, hospitals, communities, and other similar environments. The respondents were able to fill in the questionnaires on paper, or by scanning QR codes with intelligent devices; (2) online: electronic questionnaires were published on health social media platforms, and we invited some middle-aged and elderly users to fill in the questionnaires; the questionnaires were mainly distributed online, using the Qualtrics questionnaire tool. In February 2022, we sorted the data, and conducted further statistical analysis from February to March. After receiving the initial data, we first eliminated any questionnaires that appeared faulty (e.g., the answer time was too short, or the options selected were too concentrated). We conducted the following screening, based on our questionnaire design, to ensure the validity of the questionnaire responses: the respondent’s age was greater than or equal to 45 (according to the World Health Organization’s definition of middle age). Furthermore, we were seeking the opinions of respondents who had participated in HRSM at least once a week for the last year. We collected 410 online responses to the questionnaire. After manual and computer screening, 348 valid questionnaires were finally obtained (those eliminated had: answered for less than 5 min—7 questionnaires; over-centralized on the option selection—4 questionnaires; reversed two questions in the middle of the questionnaire— 51 questionnaires).

The demographic outcomes are shown in Table 2. The majority of respondents were aged between 55 and 64. The gender distribution was acceptable, with females accounting for 47.4% of the total number of subjects, and males for 52.6%. In terms of education level, the respondents to this study were generally highly educated, and the education level of a few participants (14.1%) was lower than the level of bachelor’s degree. The statistics from the numerical characteristics of the demographic variables indicated that the distribution of the investigated objects met the requirements of the survey, and that there was no significant difference in the overall characteristics of the sample.

### 3.3. Data Analysis

PLS-SEM is a multivariate statistical method that can efficiently and quickly explore complex relationships between independent variables and dependent variables. It overcomes many of the limitations of traditional linear regression analysis methods, such as large sample sizes and multiple correlation problems. Therefore, PLS-SEM was utilized in this study, together with SmartPLS 3.3.7, to verify the proposed model. Key variables, such as protection motivation, social motivation, and perceived usefulness, were set as latent variables that could not be directly observed. PLS-SEM was used to evaluate the measurement model and the structural model, and the causal relationship between variables was tested through path analysis.

**Figure 2:**
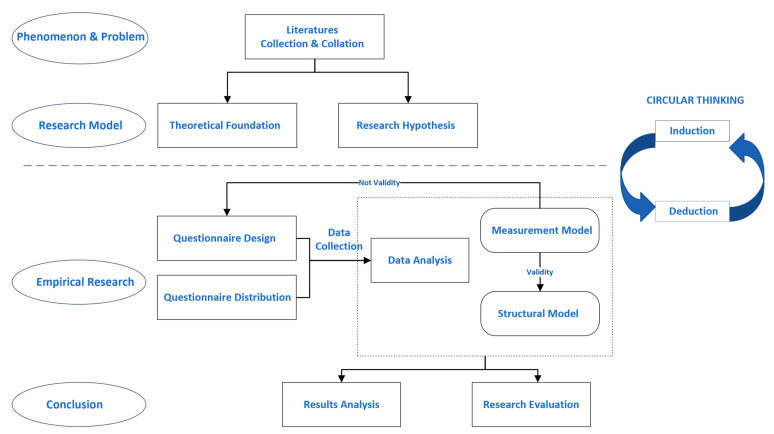
Research procedure.

The critical steps of the analysis were divided into two steps: (1) the reliability and validity of the measurement model were analyzed; the internal reliability was verified by composite reliability (CR), Cronbach’s alpha (CA), and Average Variance Extracted (AVE); then, AVE evaluated the convergent validity, and the Fornell–Larcker metric and cross-loading were used to test the discriminative validity; (2) the structural model was evaluated by the coefficient of determination (*R*^2^); meanwhile, through the *t*-value of the tested path coefficient, the research hypothesis was verified, and the model was explained. The specific experimental procedure is shown in Figure 2.

## 4. Results

### 4.1. Measurement Model Analysis

To ensure the internal consistency of the scale, we performed a reliability test, using CA, CR, and AVE. As shown in Table 3, CA was greater than 0.8, CR was greater than 0.90, and AVE > 0.80 (0.70 thresholds). All three conditions indicated that each indicator had a high level of consistency validity: that is, strong internal reliability [88].

The AVE values for all the constructs exceeded 0.8 (as shown in Table 3), which illustrated good convergence validity [89]. For discriminant validity, we used the Fornell–Larcker indicator and cross-loadings. Table 4 illustrates that the square root of the AVE was larger than the correlations between any two constructs. As shown in Table 5, the factor loadings of any index were greater than the cross-loadings of itself and the other indexes. These all indicate satisfactory discriminant validity [90].

### 4.2. Structural Model Analysis

**Figure 3:**
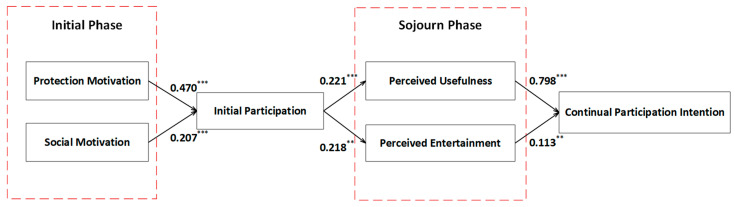
PLS-SEM analysis results. (** *p* < 0.01, *** *p* < 0.001.)

After verifying the reliability and validity of the measurement model, we then analyzed the structural model, to verify the hypotheses relationships. In this study, bootstrapping in SmartPLS was used to test the statistical significance of the t value of the path coefficient in the research model. The number of responses included was 348, and the maximum number of iterations was 5000. The path coefficient and the significance results are shown in Figure 3 and Table 6.

As shown in Figure 2, all six hypotheses proposed in this paper passed the significance test. Specifically, protection motivation and social motivation both had a significantly positive effect on initial participation in health-related social media for middle-aged and elderly people (*β*1 = 0.470, *p* < 0.001; *β*2 = 0.207, *p* < 0.001). Thus, H1 and H2 were supported. As initial participation had a positive correlation with perceived usefulness and perceived entertainment value (*β*1 = 0.221, *p* < 0.001; *β*2 = 0.218, *p* < 0.01), both H3 and H4 were accepted. In addition, both the perceived usefulness value and the perceived entertainment value positively influenced the willingness to continue participation (β1 = 0.798, *p* < 0.001; *β*2 = 0.113, *p* < 0.01), with the effect of perceived usefulness being stronger than that of perceived entertainment (*β*1 > *β*2). H5 and H6 were supported.

The determination coefficient, *R*^2^, reflects the degree to which exogenous latent variables explain endogenous latent variables; it is the most commonly used coefficient for evaluating structural models, and it is used to evaluate the predictive ability of models [91]. The value of *R*^2^ is between 1 and 0. The higher the value, the greater the predictive power. Generally speaking, when *R*^2^ is between 0.5–0.75, the explanatory ability is moderate. In this model, *R*^2^ = 0.750 explicated 75% of the variation in continual participation intention. This shows that the proposed model had good explanatory ability.

## 5. Discussion

### 5.1. Discussion of Empirical Research Results

The results show that protection motivation can significantly affect the first-time participation behavior of the middle-aged and elderly. Wang et al. [92] concluded that protection intention directly promoted people’s protection behaviors. These behaviors were defined as safe behaviors during travel, such as vaccinations and insurance. We applied protection behaviors to health behaviors associated with HRSM, based on the characteristics of the study. Our research was similar to that of Zhu, Wen, Chu, Zhang, and Liu [65], with the difference that their research focused on the behavioral impact of protection motivation on social media risk-transmission stimuli. Middle-aged and elderly consumers show differences between their actual health status and their expectations when influenced by uncertain random factors. For example, the emergence of COVID-19 hit the physical and psychological health of elderly people, who showed relatively little resistance, although to varying degrees. According to Rogers [93], people will conduct threat-appraisal and coping-appraisal when they perceive danger, and then take corresponding measures to implement self-protection behaviors. Middle-aged and older people face the pressures of death, loneliness, depression, and limited economic resources, and their behaviors, living environment, metabolic risks, and other health factors change as they transition from working age to retirement and pension. The perceived threat of health risks has a positive impact on protection motivation, which directly affects people’s use behavior [61,92]. Self-control is the ideal state for the safety behaviors produced by psychological factors when faced by the uncertainty of external variables, such as lockdowns and long distances from isolation [62]. With their increased age and social experience, middle-aged and elderly individuals have the ability to maintain or improve their health through health prevention, lifestyle, and other healthy behaviors. In response to health risks, enhancement of self-efficacy can effectively mitigate the threats, promote health, and implement healthy aging [94]. Therefore, if driven by protection motivation, the middle-aged and elderly can use HRSM to conduct safety protection behaviors, such as information searches, doctor–patient communication and seeking professional advice, and they can thereby improve their self-protection mechanism.

The findings illustrate that social motivation also has a significant and positive effect. Similarly, Wang [84] points out that social motivation can modulate people’s attitudes towards media use, and directly affect their usage behavior. Middle-aged and older adults feel limited in relation to the rest of their lives, and their goals may incline towards emotional fulfilment. Whether it is due to a lack of career planning or retirement planning, the middle-aged and elderly frequently experience a sense of loneliness, both physically and psychologically. This requires the construction of a ‘virtual environment’ to promote connection, communication, and exchange in social activities, and to make up for the lack of social interaction in reality. At the level of social interaction, social media offers interactive features, such as co-viewing, sharing, and commenting [95]. Social interactions can attract users to stay online longer, while strongly prompting users to comment, upload, etc. [96,97]. Online social interactions that break through the limitations of space and time can increase the opportunities for middle-aged and elderly individuals to communicate with the outside world, and can help alleviate the unpleasant emotions caused by a lack of interpersonal communication. In responding to this perceived need, HRSM have built an online social network for middle-aged and elderly consumers, thus increasing their social participation and reshaping their social identity. Middle-aged and elderly users will find friends with a more diverse age distribution on the internet, and interaction with other users can reduce the social loneliness of the elderly, relieve their emotional stress, and promote social connection [24,25]. Above all, as middle-aged and elderly people use HRSM, they will achieve online social interaction, which will enable them to develop and maintain human messaging and human-to-human interaction through their participation.

The results revealed that initial participation enhanced perceived usefulness and perceived entertainment. In other words, these were partially developed through HRSM participation. Users who participate in social media for health-related activities primarily aim to acquire information and entertainment, although their ultimate purpose is health [98,99]. HRSM are reliant on technology: the technology makes HRSM very different from traditional paper information, and it facilitates the collecting of information quickly, so as to grasp the latest developments, and to improve efficiency. The humanized technical design is adjusted to people’s living habits, attitudes, etc., and it renders social media more suitable for middle-aged and elderly users [100]. In addition, by using technology, health-related information on social media can be disseminated in various forms, such as text, pictures, and videos. Middle-aged and elderly people tend to receive answers to their health-related questions, that are of an acceptable quality, from HRSM [101]. HRSM can convey correct information by adjusting to users’ behavioral characteristics, to facilitate communication and decision-making [102]. In addition to health information exchange, further advantages of social media are social and emotional support, fun, enjoyment, and self-status seeking [87,97,98,99]. Middle-aged and older adults who participate in HRSM in person have access to quick and easy technology, and to content that meets their needs and recreational activities, resulting in a perception of benefit. Furthermore, the application of the acquired knowledge, advice, and so on, can help improve the health literacy of the middle-aged and elderly. The ability to self-manage their health will be developed to a certain extent. Through practice, they can acquire health prevention and health care benefits. The entertainment functions, such as interactions, ‘likes’, and sharing, not only strengthen the connection between individuals and the outside world but also play a role in ‘killing time’ and generally enriching life. The benefits of HRSM use can enhance the subjective wellbeing of middle-aged and elderly adults, thereby creating a positive cycle of positive emotions.

We also found that perceived usefulness and entertainment value positively affected the intention to continue participating in HRSM among middle-aged and older adults. These findings are in line with studies by Igbaria, Schiffman, and Wieckowski [77] and by Davis, Bagozzi, and Warshaw [78], who found that usefulness, fun, and enjoyment positively influence user attitudes and behaviors. Our study was about intention to sustain engagement: by using HRSM, middle-aged and elderly adults grasp the skills needed for Web 2.0 technology, which deepens their understanding of information, people, and society, among others. In addition, it improves electronic health literacy, and helps to improve self-efficacy and bring about certain aspects of health promotion. The fun, relaxation, and soothing emotions brought about by entertainment can relieve the mental stress of middle-aged and elderly users, and help them to fill their spare time [103]. Perceived usefulness and entertainment can lead to a certain degree of satisfaction among middle-aged and elderly adults. This is beneficial for improving their perceptions of value, thereby prompting users to engage in interaction with health topics again [86]. Participation in a closed loop creates a loop and, as a result, they prefer to continue using HRSM. Furthermore, we found that both the increasing and influencing effects of perceived usefulness were stronger than those of perceived entertainment. This may be because middle-aged and elderly adults have rich social experiences, and pay more attention to practicalities than, as they did when they were younger, to the pursuit of fun. This may also be because their acceptance and use of technology is lower than that of younger groups, so they either do not use or do not enjoy the entertainment functions during the use process [104].

The relationship and difference between the study’s results and the existing studies are explained in detail. Reviewing the overall framework of the study, the first focus was on the evolution of motivation. Most of the existing studies were based on the variables of behavioral nature, to study the participation in HRSM. For example, Gonzalez, Mitra, and Turel [41] took online health seekers as the research starting point, to explore users’ interest in using HRSM. Based on the theory of motivation, different motivations are not isolated from each other. Amabile [34] matched motivation with work, to achieve corresponding motivation. In Amabile’s research, extrinsic motivation did not destroy intrinsic motivation, but cooperated. On this basis, the motivation of each stage was divided into two types: external and internal, and the two external motivations were related to each other. For protection psychology, more attention should be paid to whether the results are helpful. Furthermore, social motivation brings certain entertainment expectations. We connect the two stages of motivation evolution through the connection between initial participation and intention to continue participation. Yang, Li, Hu, and Wang [59] studied open source software’s initial and continuous participation. The study took the two as dependent variables, to discuss the effect of word-of-mouth verse and observational learning. This study was to correlate the two, which was similar to the study of Ihm and Lee [79]. They focused on the costs and benefits of social media, and used these two perceptions as a bridge. In the context of HRSM, we also studied the motivation of initial use, to make up for the research gap on perceived usefulness and entertainment after initial use. The overall process of the study focused on the coherence of user behavior.

Therefore, by exploring the initial motivation of middle-aged and elderly groups to use HRSM, and the related factors that affected their willingness to use HRSM, this paper clarified the impact of protection motivation and social motivation on the use of HRSM by middle-aged and elderly users. The related mechanisms of intention to continue use of HRSM have theoretical and practical significance.

### 5.2. Implications

With the development of technology, the user characteristics of the middle-aged and elderly groups also continue to change. In the past, most scholars worried that the digital divide would hinder the travel and consumption of middle-aged and elderly people. Now, addiction to the internet has become a noticeable phenomenon, especially in the live broadcast industry chain. Middle-aged and older people have a specific consumption ability, and are sometimes interested in emerging things. Therefore, the investigation and cognition of their behavior needed to be further explored. HRSM provide various health information channels and resources, based on the technology platform. For middle-aged and older people, HRSM are an effective tool for self-health management; however, there are relatively few studies on using HRSM for that group; and, among the users, the proportion of that group is always less than that of young people. Hence, we used motivation evolution to reveal the evolution mechanism of continuous participation attention. A two-stage motivation analysis was used to link the intention of initial and continuous participation as the key nodes. The analysis verified that protection motivation and social motivation could stimulate the participation of potential users; it also proved that perceptions of rationality and emotion could be improved after participating in HRSM. In addition, it was an essential breakthrough of this study to link initial participation and continuous use intention through two kinds of perception—the study analyzed the dynamic monitoring of HRSM users’ thinking and behavior through motivation evolution.

#### 5.2.1. Theoretical Implications

This study enriches theory related to research on middle-aged and elderly adults. The antecedents of social media participation have received extensive attention from the academic community. However, the existing research has generally focused on younger people’s use of social media. Few studies have carried out in-depth explorations of social media use among middle-aged and elderly consumers. Adults in the middle and later periods of life differ from young adults in thought, technology use, and related behaviors. Based on this, this paper examined the impact of use motivation on users’ intentions and behaviors, from the perspectives of social motivation and protection motivation. The paper’s exploration of the motivation of the middle-aged and elderly to use HRSM provides a research paradigm for related research on the internet usage habits of middle-aged and elderly consumers. Furthermore, it promotes the development of middle-aged and elderly users’ participation in socially related internet research.

This study also fills a gap in research on health-related social media. With the continuous improvement of people’s standards of living, the concept of a healthy lifestyle has received widespread attention in societies. Most previous HRSM studies focused on participation behavior, and some studies explored initial participation and continuous participation [59,85,102,105,106]. However, users’ first use affects their subsequent behaviors to a large extent [107], so it is of critical importance to explore the influence of initial behavior on intention to continue using. In this study, the initial motivations and sojourn-stage motivations were linked with initial participation. Perceived usefulness and perceived entertainment were introduced, to explore continuous intention to use HRSM in middle-aged and elderly consumers. This enriches the research results relating to HRSM, and promotes the development of relevant theories.

#### 5.2.2. Practical Implications

The aging trend of the population has affected the ‘greying’ of social media. The growing power of middle-aged and elderly adults on social media cannot be underestimated. From a practical point of view, it is of crucial significance to explore the internet usage habits of middle-aged and elderly users. Therefore, to help HRSM understand their users better, and to utilize the positive effects of HRSM, the results of this study highlight the influence of two kinds of original motivation and two perceptions of HRSM on usage intention. Specifically, this study has the following implications, outlined below, for the middle-aged and elderly, for HRSM-related technology platforms, and for governments.

For middle-aged and elderly users, improving self-efficacy is critical. The first requirement is to improve health literacy. After obtaining health information, middle-aged and elderly users need to understand, effectively identify, and retain the information, so as to make appropriate adjustments in their health behaviors. In this way, middle-aged and elderly adults can avoid invalid information, exaggerated false information, and risky information that would negatively affect their use of health information and its perceived usefulness. The second requirement is to improve their mastery and proficiency in the technology. In particular, learning some basic skills can greatly help middle-aged and elderly people to understand and use HRSM more effectively. In addition, middle-aged and elderly adults should adjust their ideation regarding loneliness and self-discipline. Furthermore, they should use the HRSM-related entertainment functions rationally, and participate in HRSM entertainment activities according to their situation.

For the platforms, this study can help HRSM platforms identify the platform usage habits of middle-aged and elderly users better, and thus help them improve the relevance of their content, so that their design is better suited for these age groups. From the perspective of protection, the platforms need to introduce and strengthen content review mechanisms, to guide health information, health knowledge, and public opinion correctly. It is necessary to add humanized design—for example, by setting time reminders to reduce and avoid the negative health effects of excessive use. At the social level, the platforms should develop an interactive format that is more suitable for middle-aged and elderly individuals. They should attract this group to want to participate in the sharing, commenting, and disseminating of high-quality and scientific health information. For instance, they should carry out activities that have health-related content, with themes that are suited to their middle-aged and elderly users. Meanwhile, from the perspectives of the technology and information content, the platforms can increase the intention of middle-aged and elderly users to continue to participate, by providing convenient and fast technology, professional health information, humorous activities, etc.

With regard to governments, they should provide constraints and guidance to platforms from a macro perspective, and effectively control information and public opinion. They should supervise the operation of platforms through policies, laws, regulations, and other means, and establish and implement mechanisms of control. Governments can also encourage technological innovation, and commercialize the HRSM technology, through age-friendly design. Another issue to be considered is that middle-aged and elderly people are vulnerable to scams. Thus, governments should publicize and popularize HRSM, including the correct use of HRSM—for example, by providing public welfare education and training courses. On the one hand, this could help middle-aged and elderly people to establish cybersecurity in their thinking, by enhancing their cybersecurity literacy and emphasizing information-screening and usage time. On the other hand, through specific behavioral guidance, the middle-aged and the elderly could experience and master the entertainment functions available from participating in HRSM, thereby enhancing their joy in life, and helping the middle-aged and elderly integrate better into the Web 2.0 era.

### 5.3. Limitations and Future Research Directions

Our work had some limitations that could be improved on in future research. Firstly, at the data level, the data collected in the empirical part of this study all came from China, and this may have limited the generalizability of the findings to other countries, due to socio-cultural differences and variations in economic development. Future studies could conduct cross-country comparisons, to identify potential differences in pathway models, for better generalizability. Secondly, the data in this paper were cross-sectional data, and subjective. In the future, longitudinal investigation and qualitative analysis could be applied, to verify the model. Thirdly, the definition of HRSM in this study was general, and did not distinguish between different types of social media. Future research could conduct comparative research on different social media types. Fourthly, other factors, in addition to protection and social motivation, influenced initial participation. Similarly, the factors that affected the intention to continue participating were not only ‘perceived usefulness’ and ‘perceived entertainment’, but also ‘perceived risk’ and ‘perceived performance’. Future research could use theories such as self-determination theory and expectation confirmation theory, to enrich the research model. Fifthly, this study focused on the transmission and evolution of motivation. Future research could analyze protection motivation and social motivation in greater detail, from the perspective of demand; it could also explore the impact of intention on specific continuous participation behaviors. In addition, the number of samples in this paper was 384, which was more than the minimum standard for the structural equation model; however, further expansion of the sample size would effectively improve the accuracy of the evaluation. Relevant factors affecting the sustained intention of middle-aged and elderly users could also be further explored, through interviews or other methods.

## 6. Conclusions

This paper examined the impact of the motivation of middle-aged and older adults in regard to their intention to participate in HRSM. We divided the usage behavior of the middle-aged and elderly participants into two stages: the initial stage and the sojourn stage. The results showed that, in the initial stage, both protection motivation and social motivation positively affected the actual behavior of middle-aged and elderly users who participated in HRSM for the first time. The participation behaviors promoted by the two motivations enhanced the middle-aged and elderly users’ perceptions of usefulness and perceived entertainment; in addition, during the later stage, these two perceptions by these users were able to play a positive role in their motivation and intention to continue their use. Looking back at the study, in a comparison with social motivation, it was apparent that protection motivation had a more significant effect on the behavior of middle-aged and elderly consumers who were using HRSM for the first time. In addition, both perceived usefulness and perceived entertainment were linked to initial use and continuous intention, but the impact of perceived usefulness was the more significant. In the light of these findings, middle-aged and elderly groups should carry out targeted self-improvement development. HRSM researchers and practitioners could also refer to the conclusions, to guide their design development, and to further explore other motivational drivers. Governments should foster a social atmosphere, and maintain order in use, through the guidance and correct management of platforms intended for middle-aged and elderly users.

## Figures and Tables

**Table 1:** Constructs and measurement items.

Constructs and Items	Reference
**Protective Motivation (Prot)**	[65]
**Prot.1** I hope to get the necessary health information on HRSM.	
**Prot.2** I am willing to learn health skills and health-related knowledge on HRSM.	
**Prot.3** I hope to get professional health advice through HRSM.	
**Social Motivation (Soci)**	[84]
**Soci.1** I hope to meet and make people through HRSM.	
**Soci.2** I am willing to ask questions, reply and comment to participate in the interaction.	
**Soci.3** I want to get a sense of belonging.	
**Initial Participation (Init)**	[59,85]
**Init.1** I searched and browsed the health information.	
**Init.2** I participated in interactions (for example, questions, comments, answer, and discussion).	
**Init.3** I use other functions such as like and share.	
**Perceived Usefulness (Usef)**	[72,78]
**Usef.1** I think participating in HRSM can improve my health.	
**Usef.2** Participating in HRSM makes me feel relaxed and improves self-efficiency.	
**Usef.3** I think participating in HRSM help improve self-efficacy.	
**Perceived** **Entertainment (Ente)**	[86]
**Ente.1** By participating in HRSM, I feel fun.	
**Ente.2** By Participating in HRSM, I feel relieved and relaxed.	
**Ente.3** By participating in HRSM, I think I can pass time.	
**Continual Participation Intention (Cont)**	[87]
**Cont.1** I am willing to continue to use the functions of HRSM.	
**Cont.2** I would like to spend considerable time in HRSM.	
**Cont.3** I am willing to continue to participate in HRSM-related activities.	

**Table 2:** Demographic profile of respondents, N = 348.

Measure	Category	N	Percent
Gender	Male	183	52.6%
	Female	165	47.4%
Age	45–54	63	18.1%
	55–64	194	55.7%
	Over 65	91	26.1%
Education	High School and below	49	14.1%
	College	104	29.9%
	Undergraduate	143	41.1%
	Postgraduate	52	14.9%

**Table 3:** Descriptive statistics for the constructs.

Construct	CA	rho_A	CR	AVE
Cont	0.949	0.949	0.967	0.907
Usef	0.889	0.891	0.931	0.818
Ente	0.915	0.917	0.946	0.855
Inti	0.971	0.971	0.981	0.945
Prot	0.970	0.974	0.981	0.944
Soci	0.901	0.907	0.938	0.834

**Table 4:** Correlations among constructs and the square root of the AVE.

Construct	Cont	Usef	Ente	Init	Prot	Soci
Cont	**0.952**					
Usef	0.861	**0.904**				
Ente	0.554	0.553	**0.924**			
Inti	−0.028	0.221	0.218	**0.972**		
Prot	−0.599	−0.366	−0.075	0.495	**0.972**	
Soci	−0.006	0.068	0.269	0.264	0.122	**0.913**

Note: Bold numbers represent the square roots of the AVEs.

**Table 5:** Factor loadings and cross-loadings.

Construct	Cont	Usef	Ente	Init	Prot	Soci
Cont.1	**0.954**	0.830	0.576	−0.012	−0.535	0.020
Cont.2	**0.944**	0.804	0.506	−0.036	−0.574	−0.013
Cont.3	**0.958**	0.824	0.500	−0.031	−0.603	−0.025
Usef.1	0.747	**0.887**	0.500	0.234	−0.313	0.047
Usef.2	0.828	**0.928**	0.542	0.186	−0.360	0.043
Usef.3	0.757	**0.898**	0.456	0.180	−0.320	0.097
Ente.1	0.471	0.499	**0.907**	0.227	−0.038	0.282
Ente.2	0.543	0.517	**0.934**	0.190	−0.097	0.244
Ente.3	0.520	0.517	**0.931**	0.190	−0.071	0.222
Init.1	−0.023	0.209	0.194	**0.970**	0.470	0.256
Init.2	−0.038	0.214	0.202	**0.975**	0.490	0.263
Init.3	−0.020	0.220	0.239	**0.972**	0.483	0.251
Prot.1	−0.614	−0.384	−0.076	0.510	**0.972**	0.120
Prot.2	−0.575	−0.355	−0.073	0.473	**0.977**	0.098
Prot.3	−0.553	−0.326	−0.070	0.456	**0.966**	0.138
Soci.1	0.006	0.082	0.256	0.261	0.111	**0.913**
Soci.2	−0.039	0.017	0.230	0.219	0.119	**0.908**
Soci.3	0.014	0.082	0.248	0.241	0.105	**0.919**

Note: Bold numbers indicate outer loading on the assigned constructs.

**Table 6:** Path coefficient.

	T Statistics (|O/STDEV|)	*p* Values
Usef → Cont	31.971	0.000
Ente → Cont	2.813	0.005
Inti → Usef	4.905	0.000
Inti →Ente	2.733	0.006
Prot → Inti	9.031	0.000
Soci → Inti	3.395	0.001

## Data Availability

The data presented in this study are available on request from the corresponding author. The data are not publicly available due to the privacy restrictions.
